# Intravenous fluid therapy: essential components and key considerations

**DOI:** 10.1097/j.pbj.0000000000000296

**Published:** 2025-08-05

**Authors:** Catarina Silva, Pedro Marcos

**Affiliations:** a Faculty of Health Sciences, University of Beira Interior, Covilhã, Portugal; b Department of Gastroenterology, Pêro da Covilhã Hospital, Covilhã, Portugal; c Department of Medical Sciences, Faculty of Health Sciences, University of Beira Interior, Covilhã, Portugal

**Keywords:** fluid therapy, intravenous, crystalloids, colloids, components

## Abstract

**Introduction:**

Intravenous (IV) fluid therapy plays a vital role in modern medical practice, particularly in critical care management. This review aims to summarize the composition, indications, and contraindications of IV fluids, serving as a useful resource for healthcare professionals.

**Methods:**

Review of the literature published in MEDLINE using PubMed and Web of Science, between 2009 and 2024. Systematic reviews, meta-analyses, expert reviews, and guidelines were preferred for analysis.

**Results:**

IV fluids can be administered for various reasons, including resuscitation, correction of electrolyte imbalances, or more critical cases. They can be divided into 2 categories: crystalloids and colloids. Crystalloids, in turn, can be subdivided into unbalanced solutions, such as salines (0.45%, 0.9%, 3%, and 20%) and dextrose 5%, or balanced solutions, such as Ringer lactate and polyelectrolytic solutions. Colloids can be derived from plasma, such as 5% albumin, or semisynthetic, such as 4% modified fluid gelatin. Crystalloids are generally more cost-effective, have a lower risk of allergic reactions, and are more readily available than colloids. However, the use of each solution should be individualized based on the patient's specific needs and corresponding conditions.

**Conclusions:**

It is essential to have a thorough understanding of available IV fluid solutions to select the best option for each patient's condition at any given time. This review summarizes the most relevant information to guide these decisions. Future research should develop IV fluids that combine the benefits of colloids and crystalloids for safer, more personalized, and cost-effective treatments.

## Introduction

Intravenous (IV) fluid therapy is an established cornerstone of medical practice. For this reason, it is increasingly important to share and spread knowledge about the composition of various types of available fluids to ensure their proper use.^1^ Each year, over 30 million patients receive IV fluids, making fluid therapy fundamental in the management of conditions such as sepsis, hemorrhagic shock, and other life-threatening illnesses.^2^ The management of IV fluid therapy should be performed with awareness, acknowledging its potential effects on multiple organ systems. It is imperative to administer these fluids cautiously and not “blindly.”^3^

The historical significance of fluid therapy became especially apparent during the cholera epidemic of 1830, which was one of the most devastating pandemics in modern history.^4,5^ The urgent need for effective treatments prompted British physician Thomas Latta to develop the first IV therapy for cholera in 1832. This therapy consisted of a mixture of water and sodium, and it laid the groundwork for future advancements by researchers in various fields.^6^ For instance, in the late 19th century, Alexis Carrel conducted experiments with transplanted organs and designed a sodium chloride solution that resembled plasma, building on the earlier work of Latta.^7^ Following Carrel's efforts, numerous scientists, doctors, biochemists, and physiologists refined existing formulas through experiments on amphibians and mammals, all with the goal of improving on the contributions of their predecessors. Key figures in this field include Alexis Hartmann, Sidney Ringer, and Hartog Jakob Hamburger.^8^

The administration of IV fluids involves more than just following a specific prescription algorithm. It is often shaped by the beliefs and habits of the prescribing healthcare professional, but it must be tailored to each patient and their specific condition. Surveys show that many prescribers are unaware of the specific fluid and electrolyte needs of individual patients, as well as the composition of various IV fluid options available.^3^

We propose to conduct a brief review of IV fluid therapy to create a valuable resource for healthcare professionals, particularly young doctors and medical students. Among the 4 phases involved in fluid administration for a critically ill patient, this article will concentrate on the initial phase: resuscitation. This resource will outline the essential composition, indications, and contraindications of the most commonly used IV fluids. This review was conducted using studies published in MEDLINE using PubMed and Web of Science between 2009 and 2024. The search query used was as follows: (“Fluid therapy” OR “Fluid” OR “Resuscitation fluid” OR “Serum”) AND “Intravenous” AND (“Colloid” OR “Crystalloid”) AND “Composition” AND “Review.” Systematic reviews, meta-analyses, expert reviews, and guidelines were preferred for analysis. Only articles written in English and with free full text available were considered. The labels of IV fluids used at Pêro da Covilhã Hospital were also reviewed.

## Physiology of fluids

The main goal of IV fluid administration is to guarantee adequate tissue perfusion by increasing intravascular volume.^9^ IV fluids can be administered for various reasons, including resuscitation and the correction of electrolyte imbalances. However, before administering IV solutions, it is crucial to understand the compartments of body fluids in the human body. These compartments can be divided into 2 major categories: intracellular fluid (ICF), which accounts for nearly 60% of the body's total fluids, and extracellular fluid (ECF), constituting about 40%. Notably, the water content and ionic concentrations differ between the ICF and ECF: the ECF has a higher concentration of sodium, whereas the ICF is richer in potassium.^10^ The movement of fluid between these compartments generates hydrostatic pressure, driven by water, and osmotic pressure, driven by plasma proteins.^11^

More than a century ago, Ernest H. Starling observed that, under normal conditions, there is a balance in almost every capillary. The Starling principle is essential for understanding fluid dynamics within the human body. It identifies 4 key variables that influence fluid exchange across the capillary wall: capillary oncotic pressure, interstitial oncotic pressure, capillary hydrostatic pressure, and interstitial hydrostatic pressure.^12^ These Starling forces explain the balance between 2 processes: filtration, which is the movement of fluid out of capillaries, and reabsorption, the movement of fluid back into the capillaries. Both processes are crucial for maintaining homeostasis. This principle has been adapted to describe the hydrostatic and oncotic pressure gradients across the semipermeable membrane, which are the primary determinants of transvascular exchange.^13^ The average pressure at the arterial ends of capillaries is 15–25 mmHg higher than at the venous ends. This difference indicates that filtration predominates on the arterial side, while reabsorption is more common on the venous side.^14^

Starling's model has increasingly been replaced by the endothelial glycocalyx layer (EGL) model. This layer is located on the luminal surface of endothelial cells lining of blood vessels. It consists of a glycocalyx of membrane-bound macromolecules, including sulfated proteoglycans, hyaluronan, glycoproteins, and plasma proteins that adhere to this surface matrix.^15^ The main function of the EGL is to regulate permeability during the transcapillary exchange of water. In addition, it plays a crucial role in linking the permeability of the endothelial basement membrane to colloid oncotic pressure. This relationship is vital because it affects the membrane's permeability and subsequently influences transcapillary flow.^13,16^

## Types of IV fluids

IV fluids can be divided into 2 classes: crystalloids and colloids. In general, crystalloids distribute more readily to other tissues, while colloids remain in the intravascular space. When prescribing these fluids, it is crucial to consider the patient's medical history, fluid balance, vital signs, jugular venous pressure, and laboratory results, including blood counts, urea, creatinine, and electrolytes.^3^ Although IV fluid administration is generally considered safe, it does carry some risks. It is essential to monitor patients during and after the administration of IV fluids. Careful observation is needed for local reactions at the infusion site, such as thrombosis and phlebitis, and systemic reactions, including hypotension, fever, dyspnea, and itching.^17^ In addition, an overdose of fluids can lead to complications such as edema, compartment syndrome, acute respiratory distress syndrome, and dilutional coagulopathy.^18,19^.

The most relevant IV solutions available in clinical practice for fluid therapy are described in the next sections, emphasizing their composition, main indications, contraindications, and adverse events. A summary of their compositions is presented in Table 1. This article does not address some specific type of fluids, such as bicarbonate-based solutions and hydroxyethyl starch.

**Table 1:** Types and composition of IV fluids

Variables	Solutions								
	Crystalloids							Colloids	
	0.9% Saline	0.45% Saline	3% Saline	20% Saline	Ringer Lactate	Polyelectrolyte	Dextrose 5%	Albumin 5%	4% Modified Gelatin
pH	6.0	4.5–7.0	5.8	4,5–7,0	6.5	7.4	3.5–6.5	6.4–7.4	7.4
Osmolarity (mOsm/L)	308	154	1030	6844	280	295	278	309	274
Sodium (mmol/L)	154	77	513	3422	131	140		148	154
Chloride (mmol/L)	154	77	513	3422	112	98		128	126
Potassium (mmol/L)					5	5			0.4
Calcium (mmol/L)					3.7				0.4
Magnesium (mmol/L)						1.5			
Lactate (mmol/L)					28				
Acetate mmol/L						27			
Gluconate mmol/L						23			
Glucose mmol/L							252		

## Crystalloids

Crystalloids are predominantly based on a solution of sterile water with added electrolytes to approximate the mineral content of human plasma, allowing them to easily cross from the vascular space into the interstitium.^20^ Crystalloids come in a variety of formulations, from those that are hypotonic to plasma to those that are isotonic or hypertonic.^21^ When administered intravenously, these fluids predominantly distribute within the ECF compartment, given that around 20% stays in the intravascular space, while the remaining 80% moves into the interstitial space.^22^

These fluids are the most administered IV fluid because they are cheaper and are widely available, easily transportable and storable, and produce equivalent outcomes to colloid preparations.^23^ There are 2 types of crystalloids: unbalanced solutions (such as salines and dextrose 5%) and balanced solutions (such as Ringer lactate and polyelectrolytic solutions). 5% dextrose is isotonic when packaged but becomes hypotonic after infusion as the dextrose is quickly metabolized, leaving free water.^6,24^ While unbalanced solutions contain only sodium chloride, balanced solutions replace chloride anions with buffers such as lactate, acetate, or gluconate, which can be metabolized into bicarbonate or excreted.^16^ Regarding the latter, we have Ringer lactate, which is buffered with lactate, and polyelectrolyte solutions, such as Plasma-Lyte 148, which are buffered with acetate and gluconate.^20^

According to the Surviving Sepsis Campaign 2021 guidelines, septic shock represents one of the most significant challenges in health care, with a profound impact on millions of people worldwide. In this regard, it has been established that the use of crystalloid solutions is preferred due to their greater availability and the lack of demonstrated beneficial effects of colloids. It is also important to note that, among crystalloid solutions, a meta-analysis of randomized clinical trials has shown that balanced solutions offer more advantages compared with 0.9% saline, particularly by reducing mortality.^25^ Therefore, they should be the first choice in the management of a patient with sepsis.^26^

## Saline solutions

Saline-based fluids have traditionally been the standard treatment for IV volume replacement when blood or blood products are either unnecessary or unavailable.^3^ This discussion will focus on 3 types of saline solutions: 0.45% saline, which is hypotonic; 0.9% saline, which is isotonic; and 3% saline, which is hypertonic.

The 0.45% saline solution, known as half normal saline or 0.45% sodium chloride, contains 77 mmol/L of sodium and chloride. The pH varies between 4.5 and 7.0, being more frequently 5.6, and its osmolarity is 154 mOsm/L, making it hypotonic to human plasma. It can be used in the treatment of hypovolemia and extracellular hypertonic dehydration, i.e., for hydration purposes and in cases of hypernatremia. It is contraindicated in patients at risk of cerebral edema and fluid overload. It could lead to the syndrome of inappropriate antidiuretic hormone secretion (SIADH), hypotonicity, and electrolyte imbalances (namely hyponatremia), especially if administered improperly, such as being too quick.^27^

The 0.9% saline solution, known as normal saline or 0.9% sodium chloride, contains 154 mmol/L of both sodium and chloride, pH of 6.0, and osmolarity of 308 mOsm/L. Although many refer to it as “physiologic saline,” this solution is far from it. Its chloride concentration is significantly higher than that found in human extracellular fluid, which makes it supra-physiological. In addition, its osmolarity is not the same as that of human plasma, which is typically 288 mOsm/L.^11,16,28^ A 0.9% saline solution can be used to treat conditions such as hypovolemia, sodium depletion, and extracellular isotonic dehydration. It is also effective for volume resuscitation during cases of shock. In addition, this solution can serve as a vehicle or solvent for administering other medications.^29^ It is contraindicated in patients with hypernatremia, fluid overload, and certain renal conditions. It can lead to hyperchloremic metabolic acidosis when given in large volumes and is also associated with a proinflammatory state.^20,30-32^ In the renal system, normal saline may cause vasoconstriction, decreased glomerular filtration rate, and higher risks of kidney injury.^2,20,31^ In the lungs, it can contribute to interstitial pulmonary edema and endothelial injury.^33^ A study evaluating the use of balanced crystalloid versus normal saline in patients undergoing major abdominal surgery found that normal saline was linked to a higher requirement for vasopressor support.^20^ Despite these risks, 0.9% saline is the most commonly used crystalloid solution worldwide, effective for rapidly expanding blood volume in conditions such as dehydration, hemorrhage, and vomiting.^1,12,32^ In addition, owing to its acidifying properties, this fluid is an excellent first-line choice for patients with hypochloremic metabolic alkalosis—such as those with vomiting, nasogastric losses, or diuretic-induced alkalosis—as these patients are typically chloride-depleted and chloride-avid.^34^

Both 3% saline and 20% saline are hypertonic solutions widely used for managing intracranial pressure (ICP). 3% saline contains 513 mmol/L of sodium and chloride, with a pH ranging between 4.5 and 7.0, most commonly around 5.0, and an osmolarity of 1,027 mOsm/L, making it hypertonic to human plasma. 20% saline, on the other hand, has a similar pH range but a significantly higher osmolarity (6844 mOsm/L) and 3422 mmol/L of sodium and chloride. Both solutions establish an osmotic gradient that reduces cerebral edema and subsequently lowers ICP in patients with traumatic brain injury (TBI). Although several studies have compared these hypertonic solutions with mannitol, findings suggest that both therapies offer equivalent benefits, leaving the choice at the discretion of the healthcare professional.^35^ However, despite their effectiveness in ICP management, there is insufficient evidence regarding its impact on clinical outcomes to support a specific recommendation.^36^ There are some precautions when administering these fluids too quickly, particularly the onset of an osmotic demyelination syndrome, rebound increases in ICP, and acute renal failure.^16,37,38^ It is recommended that these hypertonic salines be injected through a central venous catheter to minimize the risk of peripheral vessel injury, endothelial damage, or thrombosis.^39^

## Ringer lactate

Ringer lactate (RL) is a calcium-containing balanced solution first described by Sydney Ringer and later modified by Alexis Hartmann, who introduced lactate to the original formulation.^12,40^ This solution contains 131 mmol/L of sodium, 5 mmol/L of potassium, 3.7 mmol/L of calcium, 112 mmol/L of chloride (substantially lower than in saline solutions), and 28 mmol/L of lactate.^41^ Including rapidly metabolized organic anions, such as lactate, has been shown to avoid increasing plasma acidity and results in fewer adverse effects, such as acid-base disturbances, compared with saline solutions.^11^ A significant advantage of lactate is that its concentration can be measured at the bedside, enabling better patient monitoring.^41^ In addition, Sydney Ringer observed that adding calcium improved cardiac contractility.^40^

RL has been associated with fewer renal side effects, faster clotting times, and improved clot strength.^42,43^ This solution can be used for fluid resuscitation, especially in surgical and trauma patients, and helps restore electrolyte balance. RL is often the first-line choice for patients with sepsis and acute pancreatitis, and in perioperative settings, it may reduce postoperative complications.^44-46^

However, RL may lead to a transient decrease in plasma osmolarity, which could be detrimental for patients with elevated ICP^47^ and is therefore contraindicated in individuals with TBI. Rapid infusion of this solution may also result in metabolic alkalosis and has been associated with increased apoptosis in tissues such as the bowel, liver, and lungs. This cellular damage can result in the destruction of macrophages, endothelial cells, epithelial cells, and smooth muscle cells.^19^ In hyperkalemia, RL is not contraindicated but requires careful use. Indeed, RL is preferable to 0.9% saline for its acid-base stabilizing properties and for redistributing intracellular potassium, while saline may exacerbate the condition.^47^

## Polyelectrolytic

Polyelectrolytic solutions closely resemble human plasma due to their more isotonic nature than other crystalloids.^39^ These solutions typically contain 140 mmol/L of sodium, 5 mmol/L of potassium, 1.5 mmol/L of magnesium, 98 mmol/L of chloride, 27 mmol/L of acetate, and 23 mmol/L of gluconate.^6^ Including magnesium, acetate, and gluconate represents an innovation in fluid therapy, necessitating closer patient monitoring.

Polyelectrolytic solutions have various indications and applications in clinical settings. They can be used to dilute medications commonly administered in intensive care, such as opioids, ketamine, and salbutamol.^6^ These solutions are particularly effective in correcting severe metabolic acidemia because their metabolism does not rely solely on the liver, resulting in a quicker response and a more pronounced alkalinizing effect. One significant advantage of polyelectrolytic solutions is their lack of calcium, which makes them compatible with blood and blood components.^23^ This characteristic proves to be extremely useful in situations of hemorrhagic shock that require rapid blood transfusions.^48^ Furthermore, the use of these solutions has been associated with a notable reduction in postoperative infections and the incidence of renal failure requiring dialysis.^49^

Although polyelectrolytic solutions offer various advantages, they can, in rare instances, trigger anaphylactic and hypersensitivity reactions. Moreover, these solutions may cause hyperkalemia in patients taking specific medications, including angiotensin-converting enzyme inhibitors, angiotensin II antagonists, tacrolimus, or cyclosporine.^23^ There is also a potential risk that these solutions could worsen severe metabolic alkalosis.^50^ Furthermore, the presence of acetate in these solutions may have adverse effects on patients undergoing hemodialysis or those with heart conditions, as it can decrease cardiac contractility and potentially lead to metabolic alkalosis due to its conversion into bicarbonate. Therefore, careful consideration and monitoring are crucial, particularly in at risk populations.

## Dextrose 5%

Dextrose, the D-isomer of glucose, is a simple sugar that can be dissolved in water, 0.9% saline, or 0.45% saline. This review specifically focuses on dextrose 5% (D5W) dissolved in water, which is classified as a hypotonic solution containing 252 mmol/L of glucose.^51^ Dextrose 5% is not commonly used for resuscitation because of its short half-life and inability to maintain oncotic pressure, making it more appropriate for maintenance regimens.^52^

This fluid is particularly important during the perioperative period for neonates, a high-risk group that frequently experiences hypoglycemia. If left untreated, hypoglycemia can result in permanent neurodevelopmental impairments, white matter abnormalities, and increased mortality.^37,53^ This fluid is generally not used as the primary fluid in cases of diabetic coma due to diabetic ketoacidosis or hyperosmolar hyperglycemic state. However, D5W may be introduced later in treatment once blood glucose levels start to drop to prevent hypoglycemia while continuing insulin therapy. In addition, D5W can be used to correct hypernatremia. It can provide caloric support when enteral nutrition is impossible and alternative nutritional support methods have not yet been established.^52^ Furthermore, dextrose 5% can be used as a diluent for medications and as an irrigation medium during surgeries, such as transurethral resection of the prostate.^54^

Common adverse effects include hyperglycemia and hyponatremia, which may cause osmotic diuresis and increase the risk of cerebral edema.^39,50^

## Colloids

Colloids are an alternative to crystalloids, and their use will depend on the patient's clinical status. They are suspensions of large macromolecules derived from plasma or semisynthetic sources that remain in the intravascular compartment, generate oncotic pressure, and cannot pass through a semipermeable membrane.^12,45,55^ Colloids are considered to be more effective than crystalloids as plasma volume expanders.^56^ The 2 main types are albumin and semisynthetic colloids, such as 4% succinylated modified fluid gelatin.^28^

## Albumin 5%

Albumin is the most abundant protein in human plasma, accounting for 50%–60% of total plasma proteins. It is critical in maintaining oncotic pressure and fluid balance within the vascular system. Furthermore, albumin is a carrier protein for electrolytes, hormones, and drugs; acts as a buffer for hydrogen ions; and exhibits antioxidant and scavenging properties.^52,57^

Human albumin solution is manufactured from cryo-depleted human plasma, and it is considered the safest colloid.^58,59^ Formulations include iso-oncotic solutions, such as 5% albumin, which have oncotic pressures similar to plasma, and hyperoncotic solutions, which contain less sodium and are used less frequently in clinical practice. Around 70–80% of the infused albumin stays within the intravascular space, while the remaining 20–30% moves into the interstitial space.^60^

The albumin 5% is indicated for patients experiencing marked hypoalbuminemia, peripheral edema, or those requiring fluid removal. In cases of cirrhosis, albumin infusion reduces the risk of circulatory dysfunction caused by paracentesis and lowers the risk of spontaneous bacterial peritonitis, which in turn decreases the incidence of hepatorenal syndrome.^16,57,61-63^

Although albumin has benefits, its high cost, the requirement for glass containers for distribution, concerns about viral transmission from blood-derived products, and the risk of allergic reactions (especially in patients with a tendency toward hypersensitivity) contribute to its declining popularity.^45,52^ Moreover, it is not recommended for patients with TBI, as it has been associated with increased mortality in this group^45,57,64^ and should be used with caution in patients with heart failure.

## 4% modified fluid gelatin

The high cost and limited availability of human albumin solutions have driven the development of semisynthetic colloid solutions, such as gelatins.^16^ Gelatins are produced by hydrolyzing bovine or porcine collagen. This discussion will focus on 4% succinylated modified fluid gelatin, commonly known as 4% modified fluid gelatin or Gelofusine, which contains 154 mmol/L of sodium, 120 mmol/L of chloride, and 0.4 mmol/L each of calcium and potassium.

The 4% modified fluid gelatin has a low chloride content, making it a suitable option for patients with hyperchloremic acidosis. In addition, its reduced calcium concentration enhances compatibility with blood transfusions. The lower calcium content is particularly significant because calcium serves as a vital cofactor in the coagulation cascade; thus, a decreased concentration can lead to a more pronounced state of hypocoagulation during the transfusion process. However, it is important to note that gelatins have a shorter duration of action compared with albumin, which may influence fluid management strategies in critical care settings.^65^

Although this semisynthetic colloid has its benefits, it also poses significant risks. It can lead to life-threatening anaphylaxis, which may present as cardiac arrest with ST elevation. In addition, 4% modified fluid gelatin can cause hypocoagulation, by reducing clotting factors I and VIII, as well as von Willebrand factor, impairing platelet activation and decreasing the formation of thrombin and fibrin mesh.^56,66^ Furthermore, there is an increased risk of acute kidney injury due to its accumulation in the reticuloendothelial system, leading to osmotic nephrosis-like renal lesions. These lesions may include injury to the basement membrane of epithelial cells, tubular vacuolation, and increased cell death.^45,67^

## Overview of some relevant clinical trials

A review was conducted to evaluate major clinical trials studying fluid resuscitation strategies across clinical contexts, with a primary focus on critically ill patients in ICUs—including those with sepsis, trauma, hypovolemic shock, and related conditions. Landmark trials (SAFE,^68^ CRISTAL,^69,70^ ALBIOS,^71^ PLUS,^72^ Martin et al.^73^) demonstrated no significant differences in 28-day or 90-day mortality between colloids and crystalloids, reinforcing crystalloids as the first-line, cost-effective choice. In severe sepsis, albumin supplementation failed to improve survival (ALBIOS). However, Akech et al.^74^ identified colloid advantages in children with dengue-associated septic shock.

Balanced crystalloids (SMART,^75^ SALT-ED^76^) showed superior renal outcomes over saline, particularly in reducing major adverse kidney events. Conversely, trials comparing restrictive (early vasopressor prioritization) versus liberal (high-volume fluid-first) strategies (RELIEF,^77^ CLASSIC,^78^ CLOVERS^79^) revealed that restrictive approaches lacked mortality benefits and were associated with higher complication rates (e.g., acute kidney injury).

The FENICE study^80^ highlighted that 48% of fluid challenges in ICUs were ineffective, underscoring the need for standardized protocols, objective hemodynamic criteria, and controlled volumes to avoid fluid overload. Collectively, these findings advocate for context-specific fluid selection over universal protocols. A summary is presented in Table 2.

**Table 2:** Overview of some relevant clinical trials

Author (y)	No. of patients	Clinical condition	Interventions	Main results
SAFE Study Investigators (2004) (SAFE)^68^	6,997	Critically ill adults requiring fluid resuscitation in the ICU	4% albumin vs. 0.9% saline in IVF resuscitation	No mortality difference between the 2 groups Albumin may be harmful in TBI. Saline remains a cost-effective first-line fluid, while albumin may be considered in select cases
Caironi et al (2014) (ALBIOS)^71^	1,818	Patients with severe sepsis or septic shock	20% Albumin and crystalloid solution vs. crystalloid solution alone	In severe sepsis, adding albumin to crystalloids did not improve 28-day or 90-day survival rates
Annane et al (2013) (CRISTAL)^69^	2,857	Patients with sepsis, trauma, and hypovolemic shock without sepsis or trauma	Crystalloids vs. colloids in patients admitted in ICU	In ICU patients with hypovolemia, colloids did not significantly reduce 28-d mortality compared with crystalloids. Although 90-d mortality appeared lower with colloids, this finding is exploratory and requires further investigation
Heming et al (2017) (CRISTAL)^70^	220	Acute severe hypovolemic shock in critically ill patients	Crystalloids vs. colloids in IVF resuscitation	In patients with acute severe hypovolemic shock, colloid resuscitation achieved similar hemodynamic outcomes to crystalloids but required lower fluid volumes and was associated with a lower heart rate
Akech et al (2010)^74^	Non specified	Children with shock and major infections (malaria, sepsis, dengue)	Crystalloids vs. colloids	Colloid has more advantages in dengue severe septic shock Insufficient evidence to recommend a specific fluid; further trials are needed
Finfer et al (2022) (PLUS)^72^	5,037	Patients admitted to ICU requiring fluid resuscitation	Plasma-Lyte 148 vs. saline	The 90-d mortality was similar between the BMES group and the saline group. Secondary outcomes, including renal replacement therapy and serum creatinine levels, were also comparable
Cecconi et al (2015) (FENICE)^80^	2,213	Critically ill adult patients in the ICU undergoing fluid challenges for hemodynamic assessment	Rapid infusion of fluids, predominantly crystalloids, with wide variation in volume, type of fluid, and criteria for assessing response, based on local clinical practice	While 52% of fluid challenges improved cardiac output, the remaining half proved ineffective, highlighting the critical need for evidence-based standardization of fluid protocols
Myles et al (2018) (RELIEF)^77^	3000	Patients undergoing major abdominal surgery with an expected duration of at least 2 hours and anticipated hospital stay exceeding 3 days	Restricted fluid strategy vs. liberal fluid strategy	Restrictive fluid regimen did not lead to higher disability-free survival at 1 year compared with a liberal regimen Restrictive approach was associated with increased rates of acute kidney injury, surgical-site infections, and a higher need for renal-replacement therapy
Semler et al (2018) (SMART)^75^	15,802	Critically ill adults admitted to ICU requiring IVF	Balanced crystalloids (Ringer lactate or Plasma-Lyte) vs. saline	Balanced crystalloids resulted in a lower rate of major adverse kidney events at 30 d compared with saline in critically ill adults While the absolute differences were small, the findings suggest that balanced crystalloids may be preferable to saline in this population
Self et al (2018) (SALT-ED)^76^	13,347	Noncritically ill adult patients requiring intravenous fluid therapy in the emergency department and admitted to hospital wards (not to the ICU)	Balanced crystalloids (Ringer lactate or Plasma-Lyte A) vs. saline Group	Balanced crystalloids did not result in a significant difference in hospital-free days compared with saline Balanced crystalloids were associated with a lower incidence of major adverse kidney events within 30 d
Meyhoff et al (2022) (CLASSIC)^78^	1,554	Adult with septic shock with elevated lactate (≥2 mmol/L), need for vasopressors, and recent administration of at least 1L of IV fluids in the past 24 h	Restrictive fluid strategy vs. standard care	Restrictive fluid strategy did not improve 90-d survival or reduce serious adverse events compared with standard fluid therapy
Shapiro et al (2023) (CLOVERS)^79^	1,563	Adult patients (≥18 y) with suspected or confirmed infection and sepsis-induced hypotension (systolic blood pressure <100 mm Hg after administration of ≥1,000 mL of intravenous fluids)	Restrictive fluid strategy vs. liberal fluid strategy	Restrictive fluid strategy did not result in significantly lower (or higher) mortality before discharge home by day 90 compared with a liberal fluid strategy
Martin et al (2019)^73^	11,956 (pooled population across included trials)	Critically ill adults in ICU requiring volume resuscitation	Crystalloids vs. colloids	No statistically significant difference between the 2 fluids in mortality Albumin demonstrated a safety profile comparable with crystalloids HES was associated with a higher risk of acute kidney injury and increased need for renal replacement therapy Crystalloids remain the first-line choice for fluid resuscitation due to their lower cost and favorable safety profile

BMES, balanced multielectrolyte solution; HES, hydroxyethyl starch; ICU, intensive care unit; IVF, intravenous fluid therapy; TBI, traumatic brain injury.

## Conclusions

When administering IV fluids, it is essential to consider the patient's history, ongoing monitoring parameters, and any potential complications, while being mindful of their acid-base effects. Both crystalloids and colloids have their specific uses, benefits, and risks. Careful selection and monitoring are key to optimizing fluid therapy and ensuring patient safety. This review provides a relevant and quick reference summary of IV fluid therapy.

Crystalloids, saline, and balanced solutions are well-known options. 0.9% saline solution is generally the default first choice in most clinical settings. However, a thorough analysis of the indications, advantages, and disadvantages of each fluid, alongside with a direct comparison with balanced solutions, suggests that balanced fluids, particularly Ringer lactate and polyelectrolytic solution, would be the appropriate first-line fluids for most clinical contexts. In addition, the buffering of balanced solutions with anions other than chloride provides significant advantages, such as improved ionic interactions and reduced adverse effects. Within the range of crystalloids, dextrose 5% is a fluid with more limited clinical indications compared with other options.

When comparing colloids to crystalloids, the advantages of colloids are limited. For instance, albumin, a natural colloid, is valuable in specific cases, such as in cirrhosis, post bypass surgery, and severe septic shock. However, as a blood-derived product, it poses certain risks and is expensive. This has led to the development of semisynthetic colloids, such as 4% modified fluid gelatin, which also requires careful consideration before use.

The ongoing debate regarding the use of crystalloids versus colloids for volume expansion continues. Colloids theoretically have the advantage of remaining in the intravascular space longer, which allows for better management of fluid therapy volume and more effective volume expansion.^81^ On the other hand, crystalloids are more cost-effective, carry a lower risk of allergic reactions, and are more readily available, making them the most reliable and commonly administered fluid type. However, it is mandatory to thoroughly understand available IV fluid solutions to select the best option for each patient's condition at any given time.

In conclusion, IV fluid therapy is an essential tool in managing patients. Customizing IV fluid therapy according to each patient's needs is crucial to enhance its effectiveness and reduce potential complications. This review compiles the most relevant information on IV fluid therapy for young doctors and medical students, providing the context necessary to make well-informed decisions and improve patient safety. Table 3 presents a summary of the main statements presented in this article. From a research perspective, prioritizing the development of new IV fluid formulations that combine the volume-expanding benefits of colloids with the safety and cost-effectiveness of crystalloids could be essential. The advancements in IV fluid therapy should aim toward safer, more effective, more personalized, and economically sustainable practices.

**Table 3:** Main statements

Clinicians must understand intravenous fluid options and their compositions to use them properly
It is essential to consider the patient's medical history, monitoring parameters, and possible complications, while being mindful of the acid-base effects of the chosen fluids, to make an appropriate solution selection
When comparing colloids and crystalloids, the advantages of colloids are limited
Colloids stay longer in the intravascular space but are costlier, less accessible, and riskier
Crystalloids, particularly balanced solutions such as Ringer lactate and polyelectrolytic solution, are generally appropriate first-line choices in most clinical contexts
